# Lateralized memory circuit dropout in Alzheimer’s disease patients

**DOI:** 10.1093/braincomms/fcaa212

**Published:** 2020-12-10

**Authors:** Ashley Tyrer, Jessica R Gilbert, Sarah Adams, Alexandra B Stiles, Azziza O Bankole, Iain D Gilchrist, Rosalyn J Moran

**Affiliations:** Department of Engineering Mathematics, University of Bristol, Bristol BS8 1UB, UK; National Institute for Mental Health, Bethesda, MD 20892, USA; School of Medicine, University of Virginia, Charlottesville, VA 22904, USA; College of Medicine, Ohio State University, Columbus, OH 43210, USA; Department of Psychiatry and Behavioural Medicine, Virginia Tech Carilion School of Medicine, Roanoke, VA 24016, USA; School of Psychological Science, University of Bristol, Bristol BS8 1TU, UK; Department of Neuroimaging, Institute of Psychiatry, Psychology & Neuroscience, King’s College London, London SE5 8AF, UK

**Keywords:** Alzheimer’s disease, computational psychiatry, EEG, executive function, memory

## Abstract

Altered connectivity within neuronal networks is often observed in Alzheimer’s disease. However, delineating pro-cognitive compensatory changes from pathological network decline relies on characterizing network and task effects together. In this study, we interrogated the dynamics of occipito-temporo-frontal brain networks responsible for implicit and explicit memory processes using high-density EEG and dynamic causal modelling. We examined source-localized network activity from patients with Alzheimer’s disease (*n* = 21) and healthy controls (*n* = 21), while they performed both visual recognition (explicit memory) and implicit priming tasks. Parametric empirical Bayes analyses identified significant reductions in temporo-frontal connectivity and in subcortical visual input in patients, specifically in the left hemisphere during the recognition task. There was also slowing in frontal left hemisphere signal transmission during the implicit priming task, with significantly more distinct dropout in connectivity during the recognition task, suggesting that these network drop-out effects are affected by task difficulty. Furthermore, during the implicit memory task, increased right frontal activity was correlated with improved task performance in patients only, suggesting that right-hemisphere compensatory mechanisms may be employed to mitigate left-lateralized network dropout in Alzheimer’s disease. Taken together, these findings suggest that Alzheimer’s disease is associated with lateralized memory circuit dropout and potential compensation from the right hemisphere, at least for simpler memory tasks.

## Teaser

Tyrer *et al.* used dynamic causal modelling and parametric empirical Bayes to analyse source-localized network activity in Alzheimer’s disease patients and controls. Patients showed significantly reduced left hemisphere-specific connectivity during explicit memory testing and task-associated increases in right-hemisphere connectivity, suggesting lateralized network dropout and potential right hemisphere compensation in Alzheimer’s disease.

## Graphical Abstract

**Graphical Abstract:**
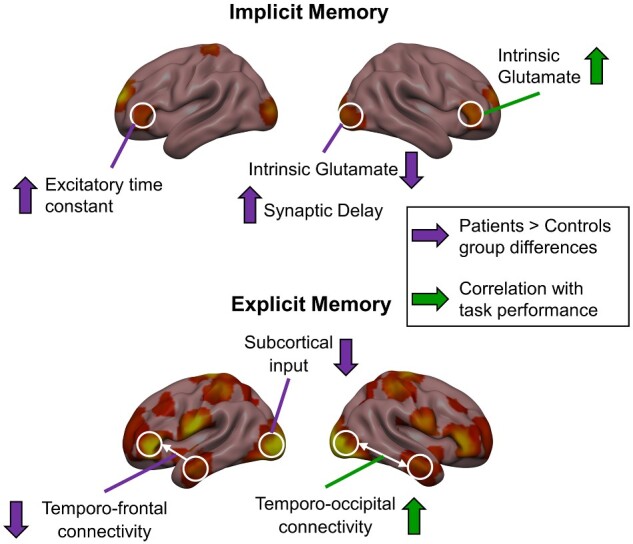

## Introduction

Alzheimer’s disease is the most prevalent cause of dementia in older adults, accounting for approximately two-thirds of dementia cases (Zhang *et al.*, 2016). Key histopathological hallmarks of Alzheimer’s disease, including extracellular amyloid-beta (Aβ) aggregates and intracellular hyperphosphorylated tau neurofibrillary tangles (Buckner *et al.*, 2005), have distinct deposition patterns that may relate to aberrant patterns of network connectivity in the brains of Alzheimer’s patients. Tau pathology is most prominent in the entorhinal cortex of the medial temporal lobes in early Alzheimer’s disease stages, then progresses outwards, with hippocampal hyper- and hypo-connections both reported features of disease progression (Marks *et al.*, 2017; Pasquini *et al.*, 2019). Aβ, distributed more broadly, may relate to effects in the default mode network (Sperling *et al.*, 2009; Palmqvist *et al.*, 2017), where both enhanced and reduced functional connections have been reported in resting-state imaging studies (Hedden *et al.*, 2009; Chang *et al.*, 2018). However, despite clear evidence for widespread disruption of neural connectivity, there are limited consistent reports of compensatory connections (Gould *et al.*, 2006). By identifying functional connections that support cognition, the development of interventions that target and bolster these regional interactions could potentially delay or ameliorate disease progression.

Recent studies have reported increased right-lateralized activity as a putative compensatory mechanism in at-risk allele carriers who have not yet developed symptoms of dementia (Han *et al.*, 2007). The putative role of right-lateralized activations as a compensatory network is supported by findings showing early asymmetric alterations in Alzheimer’s disease pathology, where cortical atrophy and deposition of Aβ have been shown to be more pronounced in left medial temporal regions (Derflinger *et al.*, 2011; Frings *et al.*, 2015). Similarly, in patients with mild cognitive impairment, left-lateralized abnormalities may predominate. For example, functional imaging markers of novelty responses in the left hippocampal formation showed a positive predictive association with subsequent cognitive decline in mild cognitive impairment patients (Miller *et al.*, 2007). Also, a recent study by Weise *et al.* (2018) examined cerebral glucose metabolism in Aβ-positive subjects with mild cognitive impairment, and showed asymmetric declines in the left medial temporal lobe compared to Aβ-negative controls, with evidence of reduced asymmetry once the disease progressed to dementia (Weise *et al.*, 2018). A recent study by Penny *et al.* (2018) used dynamic causal modelling (DCM) to investigate effective connectivity during a semantic naming task in carriers of the *PSEN1* mutation, which results in early-onset familial Alzheimer’s disease, with carriers scanned pre-symptomatically and followed for over a decade. It was found that increased effective connectivity from left medial temporal to right inferotemporal sources predicted a subsequent decline in mini-mental state examination score (Penny *et al.*, 2018).

DCM is a computational method well-suited for studying putative compensatory mechanisms, as it estimates effective connectivity both within and between sources of activity, meaning that connections are examined in the context of regional activity changes. Moreover, with DCM one can derive the way in which experimental conditions or manipulations, such as cognitive tasks, recruit specific connections. Compensatory connections have been observed using DCM for EEG in healthy older adults (Gilbert and Moran, 2016). In a study of implicit (repetition priming) memory, older adults were found to recruit prefrontal-sourced top-down connections, contrasting with younger subjects who recruited a more traditional bottom-up connectivity hierarchy with feedforward input from the early visual cortex only. During this task, the bilateral visual cortex, temporal and parietal regions, and inferior frontal cortex were included as sources of activation in the DCMs. Here, we use both an implicit memory task as well as an explicit memory task to examine changes in connectivity within this network in patients with Alzheimer’s disease. medial temporal lobe -dependent explicit (recognition) memory has been shown to be impaired in early Alzheimer’s disease (Wang *et al.*, 2014), whereas implicit memory processing has been shown to be preserved, allowing for a range of performance metrics in patients (Golby *et al.*, 2005).

In this study, we used DCM and group-level parametric empirical Bayes (PEB) analyses to investigate how inter-regional connectivity and within-region dynamics during implicit and explicit memory tasks are affected in Alzheimer’s disease. We hypothesized that hierarchical left hemisphere-specific connections may be weakened in the Alzheimer’s patient cohort compared to healthy controls. We also aimed to measure whether connections in the right hemisphere provided compensation during these memory tasks. High-density EEG and behavioural data were collected from Alzheimer’s disease patients and healthy controls. Based on our findings in the PEB analysis, we focused on the left and right-hemisphere connectivity, examining putative left-hemisphere circuit dropout and right-hemisphere compensation in Alzheimer’s disease.

## Materials and methods

### Participants

Twenty-three Alzheimer’s disease patients and 21 healthy controls (patients: mean age = 80 years, range = 68–89 years, 13 females; controls: mean age = 74 years, range = 66–91 years, 12 females) were asked to complete two mnemonic tasks while 64-channel EEG recordings were collected, preceded by a behavioural encoding phase completed prior to recording. Two patients (both females) were excluded from all analyses described below as the patients were not able to key-press independently during data collection. All control participants were free from neurological or psychiatric disorders. Patients were recruited from outpatient clinics at the Carilion Centre for Healthy Aging. Patients had a presumed diagnosis that met the diagnostic and statistical manual of mental disorders criteria for clinical Alzheimer’s disease. Study protocols were approved by the Carilion Clinic Institutional Review Board and the Virginia Polytechnic and State University.

### Tasks

**Figure 1:**
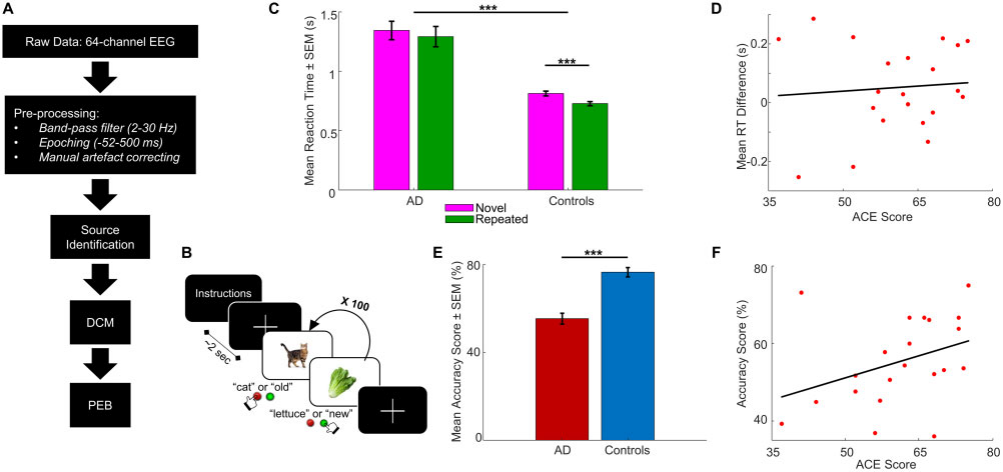
**Analysis pipeline, task structure and task performance across patients and controls.** (**A**) Schematic of the EEG data analysis pipeline, from collection and pre-processing of raw EEG data, through source identification, to constructing our DCMs and analysing our DCMs using PEB. (**B**) Visual mnemonic priming and recognition task structure. Subjects were presented with an image of an object and were instructed to covertly name the object (priming task) or indicate whether the object was old or new (recognition task), for 100 trials per task. (**C**) Mean RTs ± SEM for novel and repeated trials in the priming task, in patients and controls. Controls had significantly faster RTs across trial types than patients, and controls had significantly faster RTs in repeated trials compared to novel trials. (**D**) No correlation between ACE scores and mean RT differences for patients only in the priming task. (**E**) Mean accuracy scores ± SEM in the recognition task, in patients and controls. Controls had significantly higher accuracy scores compared to patients. (**F**) Strong correlation between ACE scores and accuracy score for patients only in the recognition task. AD = Alzheimer’s disease; SEM = standard error of mean. ***P <* *0.001.

Two separate tasks were collected during the test phase, preceded by a single encoding phase (Fig. 1B). During the encoding phase, EEG recordings were not taken. A total of 200 full-colour images were used, comprising nameable objects from well-known categories including a mix of both living and non-living stimuli (84 animals, 74 foods, 32 plants and 10 body parts). Images were presented centrally on a 1024 × 768 pixel viewing screen, were 17.8 cm × 19.1 cm (7 inch × 7.5 inch) in size, subtending a visual angle of 5°, with the longest dimension covering 300 pixels. Participants were seated ∼101.6 cm (40 inch) from the screen. In the encoding phase, participants were shown 100 images. Participants were asked to covertly name each item as quickly as possible and press the spacebar on a computer keyboard as they named each item to record reaction time (RT). Each image was presented for 2 s with a variable 1.5–2.5 s interstimulus interval in which a fixation cross was presented. After a delay period (following EEG system set-up), participants performed the priming and recognition tasks. Task order (priming versus recognition tasks) was randomized across participants. During both the priming and recognition tasks, task timing was identical to that in the encoding phase.

During the priming task, participants covertly named the 100 objects presented as quickly as possible while concurrently key-pressing to measure RT. Fifty images had not been seen before (novel) and 50 were repeated from the encoding phase (repeated), with image order randomized across participants. In line with task designs from previous picture-naming studies, covert naming was used to reduce EEG artefacts (Kan and Thompson-Schill, 2004; Gilbert *et al.*, 2010). During the recognition task, participants were again shown 100 objects, with 50 repeated from the encoding phase (but not the same repeated images used in the priming task) and 50 novel images. Participants were instructed to indicate which objects were not seen previously (novel items) and which were presented earlier (repeated items) by pressing one of two keyboard keys as quickly as possible, which were randomized across participants (Fig. 1B).

### Behavioural data analyses

During both tasks, RTs were recorded, and accuracy scores were calculated for the recognition task. Accuracy was calculated as the percentage of correct key presses (i.e. correctly identifying if the image shown was novel or repeated and pressing the correct corresponding key) of the total number of key presses in the task; missed trials were not counted towards the accuracy score.

A selection of demographic data was also collected from both patients and controls (Table 1; Supplementary material), as well as the Addenbrooke’s cognitive examination (ACE): a written neuropsychological test which examines attention, fluency, language, memory and visuospatial ability (Addenbrooke’s Cognitive Examination Revised Version, 2005) (Mioshi *et al.*, 2006). The ACE, which was initially designed as an extension of the mini-mental state examination, aims to pinpoint cognitive impairment in dementia and other neuropsychiatric conditions, including Alzheimer’s disease.

**Table 1:** Descriptive statistics for demographic data Data include number of participants (*N*), percentage of total participant number in each group (%), mean ± standard deviation and range (*N*–*N*) of demographic variables. AD = Alzheimer’s disease.

	Controls	AD patients
Participants	21	21
Female	12 (57.14%)	11 (52.38%)
Left handed	3 (14.29%)	0
Age (years)	73.71 ± 6.37 (66–91)	80.05 ± 6.18 (68–89)
ACE score	91.90 ± 4.17 (80–99)	60.86 ± 10.91 (37–75)
MMSE score	29.76 ± 0.436 (29–30)	22.62 ± 4.46 (15–30)
Education (years)	16.10 ± 2.61 (12–22)	13.19 ± 1.94 (11–18)
Social network score	7.90 ± 2.61 (4–12)	6.81 ± 3.60 (2–12)
Travel score	4.10 ± 1.34 (1–6)	2.29 ± 1.19 (0–5)
Exercise score	2.43 ± 0.507 (2–3)	1.43 ± 0.676 (1–3)
Diagnosis scan (days)		376.6 ± 720.2 (14–3192)
Depressive symptoms		5 (23.8%)
Diabetes mellitus		3 (14.3%)
Hypertension		8 (38.1%)

### EEG data acquisition and pre-processing

EEG recordings were collected using a DC amplifier (BrainAmp MR Plus, Brain Products GmbH Gilching, Germany) and a 64-channel electrode system (actiCAP, Brain Products GmbH), referenced to the average of 64 channels, as described in Gilbert and Moran (2016). Impedances of <5 kΩ for all electrodes were confirmed prior to data collection. Data were sampled at 1000 Hz and online filtered at DC-250 Hz during data acquisition.

**Figure 2:**
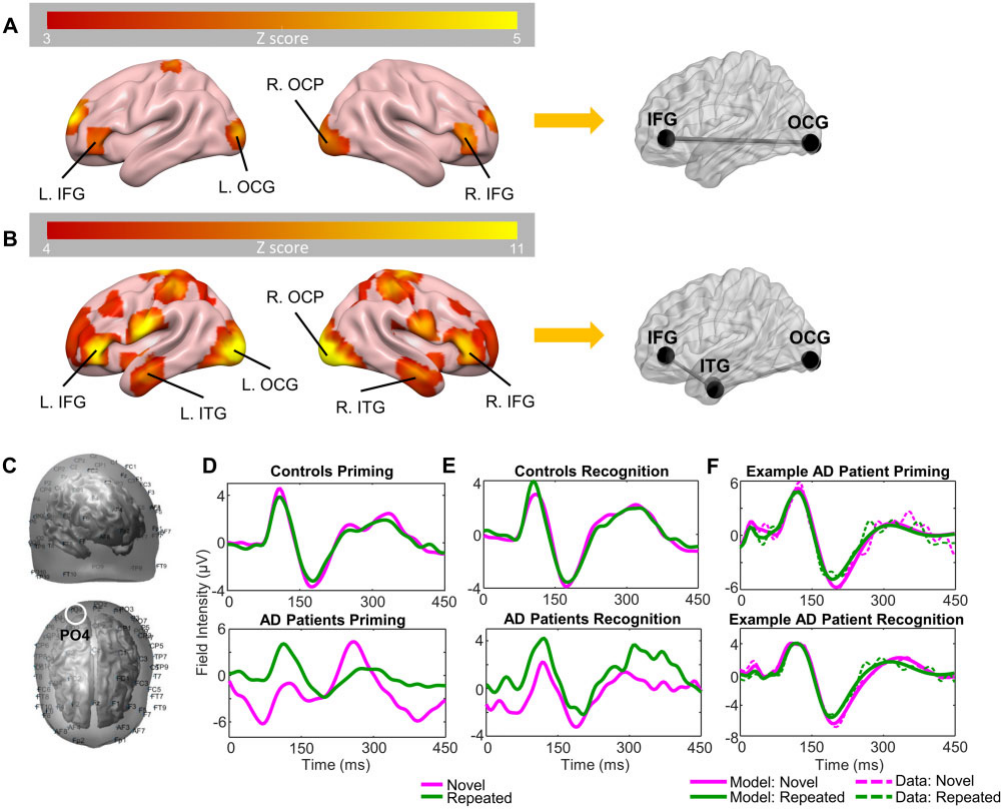
**Source identification, ERP grand means for patients and controls, and exemplary patient DCM fits.** (**A**) Bilateral four-source model identified using 3D source reconstruction for the priming task. Colour bar indicates Z scores. (**B**) Bilateral six-source model identified using 3D source reconstruction for the recognition task. Colour bar indicates Z scores. (**C**) Uncoregistered EEG sensor positions; front-right side view (top) and top view (bottom). Approximate location of channel PO4 circled in white. (**D**) Grand mean of controls (top) and patients (bottom), showing ERPs for averaged novel (magenta) and repeated trials (green) in the priming task for channel PO4 (right OCP). (**E**) Grand mean of controls and patients, showing ERPs for averaged novel and repeated trials in the recognition task for channel PO4. (**F**) DCM fits (solid line) and real data (dashed line) from the first mode of an exemplary Alzheimer’s disease patient in the priming (top) and recognition (bottom) tasks. AD = Alzheimer’s disease; L = left; R = right; MNI = Montreal Neurological Institute.

EEG data were analysed using the academic freeware SPM12 (Wellcome Trust Centre for Neuroimaging, London, UK, http://www.fil.ion.ucl.ac.uk/spm/). Pre-processing involved band-pass filtering to retain signals from 2 to 30 Hz, segmenting the continuous EEG signal into 552 ms epochs (−52 to 500 ms peristimulus time), and manually artefact-correcting to remove bad trials and channels, for example, trials containing remnant artefacts or eyeblinks. Data were then averaged based on the stimulus condition, i.e., novel images and repeated images, following baseline correction. The final pre-processed data features thus comprised event-related potentials (ERPs) over each of the 64 sensor electrodes for each condition and for each participant (see Fig. 2C for uncoregistered EEG sensor positions; Fig. 2D and E for ERP grand means). A schematic of the data analysis pipeline is shown in Fig. 1A.

### Source localization and identification

Three-dimensional spatiotemporal source reconstruction and source localization were performed using SPM’s multiple spare priors routines, to infer the network of active sources of the ERPs to inform our network model. This source reconstruction optimizes sources using a parameterized lead field, and constrained minimum norm type regression model [though constraints embody multiple (512) patches *a priori* precluding source smearing]. Sources were estimated for broadband power (2–30 Hz) over the ERP time window from 0 to 450 ms. For each participant and condition, a 3D volumetric image of sources was obtained. From these, second-level (i.e. group) analyses were performed using one-sample *t*-tests. These *t*-tests were conducted separately for the priming task and recognition task, and included both patients and controls, and both task conditions (Fig. 2A and B).

### Dynamic causal modelling

DCM served as our framework for a model-based assay of source connectivity. DCMs were specified for each individual participant to examine the modulation of extrinsic activity between patients and controls, and between the novel and repeated conditions. The DCMs were fit to the scalp-related ERPs from 0 to 450 ms peristimulus time. Based on our group-level source activity maps generated in the 3D source reconstruction analyses described above, we identified two network structures: one for each task.

### Priming network

A four-source model was used to describe the network dynamics during the implicit priming task. The sources included left inferior occipital gyrus (MNI coordinates: −32 −94 −6), right occipital pole (OCP) (MNI coordinates: 28 −96 −8), and bilateral sources in the inferior frontal gyrus pars triangularis (IFG) (MNI coordinates left: −40 40 −2 and right: 40 40 −4) (Fig. 2A), as previously reported (Gilbert and Moran, 2016).

### Recognition network

Given that the source localization results found temporal regions of activation in addition to frontal and occipital sources, we used an extended six-region network comprising occipital, temporal and frontal sources. These consisted of left inferior occipital gyrus (MNI coordinates: −30 −96 −4), right OCP (MNI coordinates: 28 −96 −8), bilateral sources in the inferior temporal gyrus (ITG) (MNI coordinates left: −46 −6 −34 and right: 46 −4 −30), and bilateral sources in the IFG pars triangularis (MNI coordinates left: −46 40 2 and right: 42 38 0) (Fig. 2B). As expected, the explicit recognition phase in the task recruited additional brain regions. Bilateral anterior temporal sources were selected for our extended explicit memory network due to their strong task relevance, in line with previous analyses (Gilbert and Moran, 2016).

We optimized DCMs for evoked responses (DCM for ERPs) for each participant individually for both models. We used a neural mass model to describe the activity at each source. Specifically, we employed the *N*-methyl-D-aspartate model (Moran *et al.*, 2011). To specify the network, we allowed for connections between these neural masses. These comprised the so-called *A* matrix (Friston *et al.*, 2003). For the priming task DCM, we specified both bottom-up and top-down hierarchical connections between the occipital sources and IFG, bilaterally for the *A* matrix, without lateral connections (Fig. 2A). For the recognition task DCM, we defined both forward and backward connections from occipital sources to ITG, and ITG to IFG bilaterally for the *A* matrix, without lateral connections (Fig. 2B). For both tasks, we assumed that no crosstalk between hemispheres would occur via lateral connections.

We then defined the *B* matrix: a connectivity matrix similar to the *A* matrix which defines task-dependent modulatory connections, i.e., the difference in novel versus repeated image trials on specified connections. For our models, we defined the *B* matrices with the same connections as in the *A* matrices, but with added self-connections for all sources. The input vector *C* defines the activity sources receiving subcortical sensory input, which here were the left and right occipital sources in the models for both tasks. The models also included parameters describing local glutamate connectivity (*G*), the time constant of post-synaptic responses (*T*) and delays between sources (*D*) (Supplementary Table 1). These parameters constitute a multivariate set θ.

These generative models of interacting sources were inverted according to a variational Bayesian scheme to examine the likelihood of parameters, given the model and data for each participant individually (Friston, 2002). Inversion of the models was performed for each task, and each subject, individually. This approximates the posterior probability of model parameters p(θ|y,m), i.e., the probability of the model parameters given the data and the model. The ERP scalp response is represented by y, and m represents the model, i.e., which regions in the brain are connected and how these connections are modulated by the tasks; θ represents the model parameters (see Fig. 2F for example patient DCM fits). For the priming task, the model had 29 parameters, and for the recognition task, the model had 43 parameters (due to the increased number of sources in the network). Given these inferred parameter sets, we next sought to determine those parameters associated with task performance, and those associated with the disease *per se*. To study these group effects, these posterior parameters were then passed into our PEB analysis outlined below.

### PEB and classical analyses

**Figure 3:**
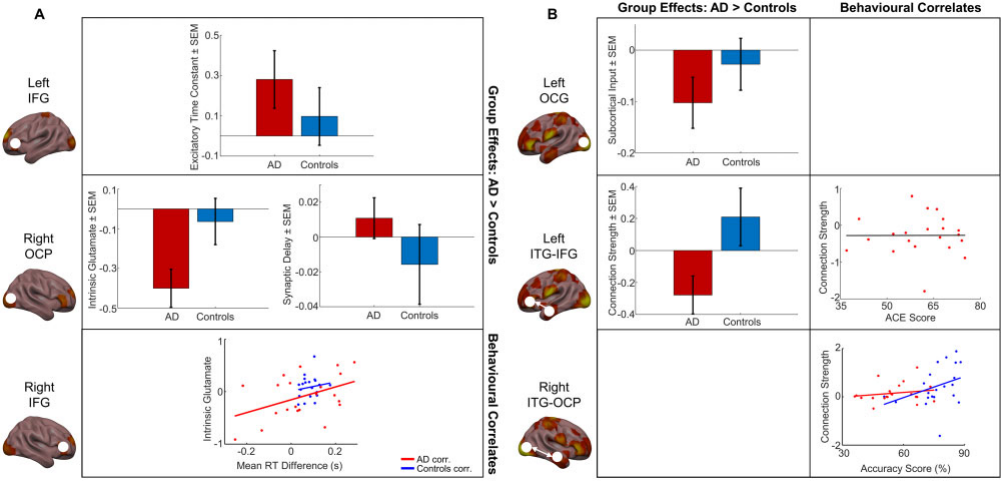
**Model parameters estimated using PEB, for group-level differences and effects of task performance.** (**A**) PEB findings in the priming task. Top: group differences in excitatory time constant between patients and controls in the left IFG, showing mean ± SEM of parameter estimates across participants. Centre: group differences in intrinsic glutamate (centre left) and synaptic delay (centre right) between patients and controls in the right OCP. Bottom: correlation between intrinsic glutamate and implicit memory task performance in the right IFG. Patients: red; controls: blue. (**B**) PEB findings in the recognition task. Top left: group differences in subcortical input into the left occipital gyrus between patients and controls, showing mean ± SEM of parameter estimates across participants. Centre left: group differences in forward connectivity strengths from the left ITG to left IFG between patients and controls, showing mean ± SEM of parameter estimates across participants. Centre right: no significant correlation between forward connectivity strengths from the left ITG to left IFG, and ACE scores in patients only (*rho* = 0.0039, *P *=* *0.987, Spearman’s rank correlation). Bottom right: correlation between backward connectivity strengths from the right ITG to right OCP, and explicit memory task performance. Patients: red; controls: blue. AD = Alzheimer’s disease; corr. = correlation; SEM = standard error of mean.

PEB was used for a random-effects analysis over model parameters, based on the presence or absence of Alzheimer’s disease and task performance for both tasks separately. The PEB comprises a Bayesian General Linear Model (GLM) at the second level. Here we constructed the Bayesian GLM, using two second-level covariates as well as including an average mean effect. A random-effects design matrix was generated containing three separate columns, one for each covariate: the first column was the average over all subjects (a column of ones), the second column defined disease, i.e., patient or control (1 or 0 respectively), and the third column defined the parametric task performance (either RT difference for the priming task, calculated as the mean novel RT minus mean repeated RT for each participant, or accuracy score for the recognition task). From this one can compute which parameters show group-level differences based on disease state (patients versus controls), and which are also affected by task performance, as well as their probabilities. Thus, the GLM allows us to examine the network correlates of task performance while accounting for disease state. We describe which connections in our models were strengthened/weakened as a result of disease, the directionality of such connections, and whether this was specific to a particular hemisphere. Also, these analyses inform us about whether such connections are modulated by task performance, and whether these connections may be performing compensatory roles in patients based on task performance. The PEB analysis essentially re-estimates model parameters at the level of individual DCMs by conducting a search over all possible parameter combinations that emulate the design matrix. The final analyses report the effect size, direction, and probability. This approach aims to reduce the second-level effects using Occam’s razor until only meaningful parameters that contribute to group differences remain (Fig. 3).

### Statistical analyses

A two-way mixed-effects ANOVA and Wilcoxon signed-rank tests were conducted to examine RT differences across patients and controls in the priming task, and a one-way ANOVA was conducted to examine differences in accuracy scores between patients and controls in the recognition task. A MANOVA was also conducted to investigate group differences in demographic variables, comparing patients and controls. These statistical tests were conducted using IBM SPSS Statistics 24 software. Spearman’s rank correlations were conducted using MATLAB software to examine associations between ACE scores and mean RT difference/accuracy scores.

Following the PEB, we also conducted *posthoc* classical statistical tests using MATLAB software on group differences in parameter values between patients and controls using two-tailed two-sample *t*-tests, and correlations between specific parameter values and the behavioural measures calculated previously (i.e. mean RT difference for the priming task and accuracy score for the recognition task) using Pearson’s correlation.

### Data availability

The data that support the findings of this study are available from the corresponding author, upon reasonable request.

## Results

### Controls consistently outperform patients in both implicit and explicit memory tasks, with high variability in patients’ task performance

We conducted a two-way mixed-effects ANOVA on the RTs in novel and repeated trials in patients and controls. This revealed a significant between-subjects main effect of disease [*F*(1,40) = 44.4, *P *<* *0.001, *η_p_*^2^ = 0.526] indicating that RTs were significantly higher in patients compared to controls. There was also a significant within-subject main effect of trial type [*F*(1,40) = 16.7, *P *<* *0.001, *η_p_*^2^ = 0.294], suggesting that RTs in novel trials were overall significantly higher than RTs in repeated trials (Fig. 1C). However, the disease × trial-type interaction was not significant [*F*(1,40) = 1.02, *P *=* *0.318], indicating that the RT differences in novel versus repeated trials did not differ significantly between patients and controls (Fig. 1C). This suggests that implicit memory may be preserved in patients in the priming task, as well as in controls.

For RTs in both novel and repeated trials, however, the variances were unequal for patients compared with controls, which may result in inflated *P* values [novel: *F*(1,40) = 28.0, *P *<* *0.001; repeated: *F*(1,40) = 30.7, *P *<* *0.001; Levene’s test of equality of error variances]. Therefore, to supplement the ANOVA, we conducted Wilcoxon signed-rank tests to examine RT differences between the novel and repeated trials in patients and controls separately. In controls, the median of novel RTs was significantly higher than that of repeated RTs (*Z* = −4.02, *P *<* *0.001). In patients, the medians of novel and repeated RTs were not significantly different (*Z* = −1.48, *P *=* *0.140).

To assess how task performance relates more broadly to cognitive decline, we examined the relationship between performance during this task and the ACE. There was no correlation between ACE scores and mean RT difference (the difference between mean novel and mean repeated RT) for patients only (*rho* = 0.0741, *P *=* *0.750) (Fig. 1D).

Task performance was measured in the recognition task by calculating accuracy scores, i.e., the number of successful responses out of total responses in the task. Behaviourally, accuracy scores for the recognition task were significantly higher for controls (mean = 0.765, SEM = 0.0210) than for patients (mean = 0.553, SEM = 0.0249) [*F*(1,40) = 42.1, *P *<* *0.001] (Fig. 1E). Once again, we investigated the relationship between ACE scores and task performance, in this case, recognition accuracy scores. There was a moderate correlation between accuracy scores and ACE scores for patients (*rho* = 0.434, *P *=* *0.0492), with high variability in the spread of accuracy scores and ACE scores (Fig. 1F).

Overall, our group-level results showed the typical decline in mnemonic processing seen in patients with Alzheimer’s disease. Moreover, we also observed high variability in accuracy scores and priming performance (i.e. RT), where variability in the explicit task was related to established clinical scales. We therefore aimed to understand how this variability is related to network connectivity using our DCMs.

### Years of education, travel and exercise scores are significantly lower in patients, but no effect of social network scores

A MANOVA was conducted to examine differences between patients and controls across the following demographic variables: years of education, travel score, social network score and exercise score (Table 1). Using Pillai’s trace, we found a significant effect of disease state, in that the demographic variables tested were significantly different between patients and controls [*V *=* *0.606, *F*(4,37) = 14.2, *P *<* *0.001]. Separate univariate ANOVAs on each demographic variable revealed significant effects of years of education [*F*(1,40) = 16.8, *P *<* *0.001], travel score [*F*(1,40) = 21.5, *P *<* *0.001] and exercise score [*F*(1,40) = 29.4, *P *<* *0.001], but did not reveal a significant effect of social network score [*F*(1,40) = 1.28, *P *=* *0.265].

The travel score contained a historical element, as participants were asked if they had previously lived abroad during their lives, suggesting that the amount of travelling an individual did during their life may influence their susceptibility to Alzheimer’s disease in later life. In contrast to the travel score, the social network score only considered each participant’s current number of social networks, rather than historical social networks prior to diagnosis, and therefore cannot be used as an accurate indication of the role of social networks in the risk of developing the disease.

Demographic variables such as years of education, travel score, and exercise may have an impact on the likelihood of an individual developing dementia later in life, however, historical data for exercise levels would be required for this to be conclusive. Rather than solely the travel scores and years of education directly affecting the susceptibility of an individual to suffering from dementia, it is more likely that these factors play roles in a complex socioeconomic interaction with additional factors.

### Patients show within-region slowing in implicit memory network

In our PEB analysis, we first tested whether there was a group difference in the connectivity strengths and modulated connectivity strengths between patients and controls, and then examined the effects of mnemonic task performance, while accounting for effects of disease state.

Controls had significantly increased strengths of intrinsic glutamate connectivity (*G*) in right OCP compared to the patients in the priming task [Effect size (*Ep*) = −0.482, posterior probability (*Pp*) = 1.00] (Fig. 3A). Additionally, patients had significantly reduced aggregate excitatory receptor activity (i.e. increased excitatory time constant *T*) in the left IFG (*Ep* = 0.367, *Pp* = 1.00) and a greater delay in signal transmission (*D*), i.e., the time taken for signals to transmit from region-to-region including axonal delays (*Ep* = 0.194, *Pp* = 1.00), indicating a general slowing in memory processing in patients. The PEB analysis also found significant associations between intrinsic glutamate connectivity in right IFG and RT difference, in that this connectivity was increased for larger RT differences (*Ep* = 1.52, *Pp* = 1.00), suggesting that task-related increases in local glutamate connectivity in the right hemisphere may have a modulatory effect on implicit memory task performance in patients only (Fig. 3A).

### Left hemisphere circuit deficits in explicit memory sub-network in patients, with task-associated connectivity increases in right hemisphere

In the recognition task, the PEB showed increased forward connectivity (*A* matrix) from left ITG to left IFG in controls compared to patients (*Ep* = −0.352, *Pp* = 1.00) (Fig. 3B). The subcortical input (*C*) into left inferior occipital gyrus was also increased in controls compared to patients (*Ep* = −0.177, *Pp* = 1.00), indicating that patients may suffer from reduced visual input into their left-hemisphere memory circuit (Fig. 3B). Top-down connections from right ITG to right OCP were found in the PEB analysis to have a significant correlation with accuracy score (Fig. 3B), as these connections increased with higher accuracy scores (*Ep* = 0.670, *Pp* = 1.00), in addition to the recurrent bottom-up connectivity from right OCP to right ITG showing similarly strong positive associations with accuracy score (*Ep* = 0.553, *Pp* = 1.00). This implies a strong association between increased right-hemisphere connectivity and improved task performance in this more taxing memory recall task while accounting for group differences.

### *Post* *hoc* classical analyses confirm PEB findings, and reveal that association between implicit memory performance and intrinsic glutamate activity is patient-driven

To confirm our findings from the PEB analysis, we then used classical statistics to further interrogate the effects of disease and task performance on the above parameter estimates.

Classical inference on these parameter estimates confirmed that in the priming task, controls had significantly increased intrinsic glutamate connectivity in right OCP compared to patients [*t*(40) = 2.22, *P *=* *0.0319], as seen in the PEB (Fig. 3A). Also, intrinsic glutamate connectivity within right IFG showed a significant positive correlation with the RT difference (*rho* = 0.421, *P *=* *0.00550) (Fig. 3A). However, this correlation is primarily driven by patients (patients only: *rho* = 0.439, *P *=* *0.0465), rather than the controls (controls only: *rho* = 0.167, *P *=* *0.468), implying that this glutamate connectivity in the right hemisphere may be playing a task-related compensatory role specifically in Alzheimer’s disease patients.

In the recognition task, classical inference on parameter estimates displayed an increase in forward connectivity from left ITG to left IFG in controls compared to patients [*t*(40) = −2.26, *P *=* *0.0295] (Fig. 3B). Furthermore, backward connectivity from right ITG to right OCP showed significant positive correlation with recognition accuracy score (*rho* = 0.354, *P *=* *0.0216), further confirming our findings in the PEB analysis of left-hemisphere dropout in patients, and task-related increases in right-hemisphere connectivity in explicit memory processing (Fig. 3B).

## Discussion

While previous studies have shown left hemisphere-specific effects in patients with Alzheimer’s disease at rest (Scahill *et al.*, 2002; Miller *et al.*, 2007), here we used DCM of task-based EEG and PEB to demonstrate that in simple priming memory tasks, Alzheimer’s disease patients suffer from slowing of implicit memory processes in the left hemisphere but display task-related right hemisphere-specific upregulation of local glutamate connectivity, which may play a compensatory role in implicit memory circuits. In the more taxing explicit memory task, we found source-level memory circuit dropout in the left hemisphere of patients which were preserved in the priming task. Also using PEB, we showed task-associated increases in connectivity strengths and local excitation specifically in the right hemisphere, implying compensatory mechanisms are being performed by the right hemisphere in these patients. Importantly, the PEB analyses enabled us to examine both group effects and the effects of each task while accounting for disease state.

We further showed that the tasks used in this study are effective in testing implicit and explicit memory, as controls consistently showed significantly better performance compared to patients as expected. Our behavioural results revealed a high level of variability in task performance of Alzheimer’s patients for both tasks; task performance was quantified as mean RT difference in the priming task and accuracy score in the recognition task. Recognition accuracy scores correlated strongly with patients’ ACE scores, an established clinical score that can be used to indicate the presence of dementia and disease progression. This correlation suggests a direct relationship between patients’ explicit memory and the severity of cognitive deficits.

These two behavioural tasks examined distinct types of memory recall: implicit priming memory and explicit recognition memory. Previous studies have demonstrated a slight preservation of implicit memory in patients with mild Alzheimer’s disease using similar priming and recognition memory tasks, specifically using picture stimuli. A study by Deason *et al.* (2015) showed preserved implicit conceptual priming memory in subjects suffering from mild Alzheimer’s disease comparing the priming effect when pictures and words were presented as visual stimuli. They found intact priming in Alzheimer’s disease patients only when pictures were used as stimuli, and also used an explicit recognition memory task to demonstrate a decline in recognition memory in patients with mild Alzheimer’s disease compared to healthy aged controls (Deason *et al.*, 2015). Another study by Martins and Lloyd-Jones (2006) showed similar effects using a fragmented picture paradigm, demonstrating preserved perceptual closure in Alzheimer’s disease patients (Martins and Lloyd-Jones, 2006). Our patient group suffered from mild to moderate Alzheimer’s disease and displayed varying severities of cognitive decline, reflected by a wide range of clinical ACE scores. This variation in the cognitive ability within the patient group could explain why, although patients were slightly slower in novel trials compared with repeated trials, this preservation of implicit memory is not statistically significant.

Using source localization, we identified distinct networks in each task: a six-source bilateral network in the recognition task; and a simplified four-source network in the priming task. These networks included left and right occipital sources and left and right frontal sources, with the addition of left and right temporal sources in the recognition task only. We expected to identify a more complex network for the recognition task, in that there is greater recruitment of medial temporal regions in explicit memory recall, as compared to priming. It naturally follows, therefore, to include bilateral ITG sources here as an extension of the network used for the priming task. As in all DCM studies, our findings are dependent on our selection of sources included in the models, however, our source selection is justified and well-supported by source localization analyses and previous work (Gilbert and Moran, 2016). Our six-source network represents a sub-network of the full explicit memory network which may employ additional regions. However, to reduce model complexity and prevent over-fitting of the model, we selected a maximum of six sources in our sub-network. The deposition of tau neurofibrillary tangles has been reported to initiate in the medial temporal lobe and spread outwards as the disease progresses (Marks *et al.*, 2017; Pasquini *et al.*, 2019), and studies have shown that Aβ has increased deposition in the left medial temporal lobe during early Alzheimer’s disease (Frings *et al.*, 2015). We then generated our DCMs for the tasks using these two different networks and conducted PEB analyses to examine both group effects and task performance.

Our PEB analyses revealed a slowing of signal transmission generally and with further slowing (increased time constants) specifically in the left hemisphere of Alzheimer’s patients. This was observed in the priming task, along with strong associations between local within-region glutamate connectivity (*G*) and task performance in the right frontal lobe, specifically the right IFG. This correlation was predominantly driven by patients only, revealed by our *posthoc* classical analyses—indicating compensatory, right-hemisphere recruitment. The spread of parameter values for glutamatergic local connectivity was much greater in patients and showed a strong correlation with the mean RT difference (i.e. task performance) in the priming task, whereas in healthy controls the range of *G* parameter values was much narrower and did not correlate with task performance, exhibiting a low variability relative to patients. This glutamate connectivity, or gain, also showed group-level differences, not related to task performance *per se*: with enhanced gain in the right OCP which was significantly greater in healthy controls compared to patients in the priming task. These findings suggest that intrinsic glutamate connectivity in the right hemisphere may act as a compensatory mechanism in Alzheimer’s patients while performing simpler implicit memory tasks, but controls have overall higher levels of this connectivity as a group and are still able to outperform patients.

In the recognition task, however, we see much larger-scale network effects between patients and controls. There was significant network dropout in the left hemisphere: forward connectivity from the left ITG to left IFG was reduced in patients compared with controls, and subcortical input into the left inferior occipital gyrus was significantly reduced in patients. This therefore indicates that left-hemisphere memory circuits are compromised in patients, and in the more difficult explicit memory task patients have a reduced capacity to compensate for this loss, as seen in their significantly reduced task performance versus controls. It may be that these networks are preserved in implicit memory processing in patients, at least early in the disease, as this dropout is absent from our PEB analysis of the simpler priming task. In terms of task-based effects, we observed a strong relationship between recurrent right-hemisphere connectivity, namely forward right OCP to right ITG and backward right ITG to right OCP, and recognition accuracy score across participants, with a higher PEB effect size for the backward connections. Thus, this right-hemisphere region-to-region connectivity may play a role in explicit memory recall.

Many studies have found that, at rest, patients suffering from mild cognitive impairment or early stages of Alzheimer’s disease have lateralized atrophy specifically in the left hemisphere (Miller *et al.*, 2007), and a resting-state MRI study by Thompson *et al.* (2001) found increased left-hemisphere grey matter atrophy in patients with mild to moderate Alzheimer’s disease compared to healthy elderly controls (Thompson *et al.*, 2001). Another resting-state MRI study (Fox *et al.*, 1996) showed significant asymmetry in the left and right hippocampal formation of pre-symptomatic individuals at risk of familial Alzheimer’s disease who were followed for 3 years and later developed symptoms of Alzheimer’s disease. The right hippocampal formation showed no significant differences to that of controls, whereas left hippocampal formations were significantly smaller than that of controls during this pre-symptomatic period (Fox *et al.*, 1996). Here, we show specific left-lateralized slowing and depletion of connectivity in patients with Alzheimer’s disease in two different memory tasks, with potential task-related compensation for implicit memory circuits in the right hemisphere.

A potential limitation of our paradigm is the use of covert rather than overt naming during the priming task. However, the explicit identification of an object during priming does not affect long-term object priming as shown in a recent study (Gomes and Mayes, 2015), and whether subjects were consistent in their naming of objects is of greater importance than the particular name given to the object in the context of the priming task.

The use of EEG in this paradigm was essential for estimating parameters such as excitatory time constants and signal delays. EEG delivers a high temporal resolution at timescales constant with that of synaptic transmission; this is in contrast with other human neuroimaging techniques such as fMRI. While fMRI may offer much more powerful spatial resolution, in our experiment it was crucial that we obtain more temporally resolved, direct measures of neuronal activity in order to scrutinize parameters inferred using our DCMs which span from macroscale region-to-region connectivity, to mesoscale ensembles of cellular and synaptic dynamics.

Future work may examine potential lateralized compensatory mechanisms and cognitive reserve in bilingualism. Bilingualism has been widely reported to delay the onset of many forms of dementia, including Alzheimer’s disease (Bialystok *et al.*, 2007; Craik *et al.*, 2010). Bilingual brains have also been shown to undergo experience-associated neuro-structural alterations, particularly in left-hemispheric regions such as the left IFG (Stein *et al.*, 2012) and left inferior parietal lobule (Della Rosa *et al.*, 2013). These changes may be neuroprotective in age-related cognitive decline as compared with brains of monolinguals (Abutalebi *et al.*, 2014). Bilingual brains may therefore be able to better compensate the loss of connectivity in the left hemisphere that is observed in dementias such as Alzheimer’s disease. Such work could offer powerful insights into compensatory and neuroprotective mechanisms against Alzheimer’s disease. Overall, our results speak to a relative specificity of functional pathology in regional circuit-level signal integration and how compensatory measures may be identified.

## Supplementary material

Supplementary material is available at *Brain Communications* online.

## Funding

A.T. is supported by the Wellcome Trust PhD programme in Neural Dynamics. This work was supported by a Virginia Tech Carilion Research Institute start-up grant to R.J.M.

## Competing Interests

The authors report no competing interests.

## Supplementary Material

fcaa212_Supplementary_Data

## Abbreviations

Aβ =: amyloid-beta
ACE =: Addenbrooke’s cognitive examination
DCM =: dynamic causal model
Ep =: effect size
ERP =: event-related potential
GLM =: general linear model
IFG =: inferior frontal gyrus pars triangularis
ITG =: inferior temporal gyrus
MMSE =: mini-mental state examination
MTL =: medial temporal lobe
OCP =: occipital pole
PEB =: parametric empirical Bayes
Pp =: posterior probability
RT =: reaction time
SPM =: statistical parametric mapping
