# Supplementary material for: Lateralized memory circuit dropout in Alzheimer’s disease patients
Source: Brain Commun. 2020 Dec 10;2(2):fcaa212. doi: 10.1093/braincomms/fcaa212 (PMC7772115; doi:10.1093/braincomms/fcaa212)
Supplement: fcaa212_Supplementary_Data [file fcaa212_supplementary_data.pdf]

Supplementary Table 1A: PEB parameters in the priming task

Parameter	Parameter Description
S(1)	Overall excitability
T(1)	AMPA time constant left iOCG
T(2)	AMPA time constant right OCP
T(3)	AMPA time constant left IFG
T(4)	AMPA time constant right IFG
G(1)	Intrinsic glutamate connectivity within left iOCG
G(2)	Intrinsic glutamate connectivity within right OCP
G(3)	Intrinsic glutamate connectivity within left IFG
G(4)	Intrinsic glutamate connectivity within right IFG
A{1}(3,1)	Forward connections from left iOCG to left IFG
A{1}(4,2)	Forward connections from right OCP to right IFG
A{2}(1,3)	Backward connections from left IFG to left iOCG
A{2}(2,4)	Backward connections from right IFG to right OCP
B{1}(1,1)	Trial-dependent self-connections of left iOCG
B{1}(3,1)	Trial-dependent connections from left iOCG to left IFG
B{1}(2,2)	Trial-dependent self-connections of right OCP
B{1}(4,2)	Trial-dependent connections from right OCP to right IFG
B{1}(1,3)	Trial-dependent connections from left IFG to left iOCG
B{1}(3,3)	Trial-dependent self-connections of left IFG
B{1}(2,4)	Trial-dependent connections from right IFG to right OCP
B{1}(4,4)	Trial-dependent self-connections of right IFG
C(1)	Subcortical input into left iOCG
C(2)	Subcortical input into right OCP
R(1)	Stimulus onset parameter
R(2)	Stimulus dispersion parameter
D(1)	Within-region cell-to-cell population signal delay
D(2)	Between-region signal delay
U(1)	Exogenous background activity
CV(1)	Membrane capacitance

Supplementary Table 1B: PEB parameters in the recognition task

Parameter	Parameter Description
S(1)	Overall excitability
T(1)	AMPA time constant left iOCG
T(2)	AMPA time constant right OCP
T(3)	AMPA time constant left ITG
T(4)	AMPA time constant right ITG
T(5)	AMPA time constant left IFG
T(6)	AMPA time constant right IFG
G(1)	Intrinsic glutamate connectivity within left iOCG
G(2)	Intrinsic glutamate connectivity within right OCP
G(3)	Intrinsic glutamate connectivity within left ITG
G(4)	Intrinsic glutamate connectivity within right ITG
G(5)	Intrinsic glutamate connectivity within left IFG
G(6)	Intrinsic glutamate connectivity within right IFG
A{1}(3,1)	Forward connections from left iOCG to left ITG
A{1}(4,2)	Forward connections from right OCP to right ITG
A{1}(5,3)	Forward connections from left ITG to left IFG
A{1}(6,4)	Forward connections from right ITG to right IFG
A{2}(1,3)	Backward connections from left ITG to left iOCG
A{2}(2,4)	Backward connections from right ITG to right OCP
A{2}(3,5)	Backward connections from left IFG to left ITG
A{2}(4,6)	Backward connections from right IFG to right ITG
B{1}(1,1)	Trial-dependent self-connections of left iOCG
B{1}(3,1)	Trial-dependent connections from left iOCG to left ITG
B{1}(2,2)	Trial-dependent self-connections of right OCP
B{1}(4,2)	Trial-dependent connections from right OCP to right ITG
B{1}(1,3)	Trial-dependent connections from left ITG to left iOCG
B{1}(3,3)	Trial-dependent self-connections of left ITG
B{1}(5,3)	Trial-dependent connections from left ITG to left IFG
B{1}(2,4)	Trial-dependent connections from right ITG to right OCP
B{1}(4,4)	Trial-dependent self-connections of right ITG
B{1}(6,4)	Trial-dependent connections from right ITG to right IFG
B{1}(3,5)	Trial-dependent connections from left IFG to left ITG
B{1}(5,5)	Trial-dependent self-connections of left IFG
B{1}(4,6)	Trial-dependent connections from right IFG to right ITG
B{1}(6,6)	Trial-dependent self-connections of right IFG
C(1)	Subcortical input into left iOCG
C(2)	Subcortical input into right OCP
R(1)	Stimulus onset parameter
R(2)	Stimulus dispersion parameter
D(1)	Within-region cell-to-cell population signal delay
D(2)	Between-region signal delay
U(1)	Exogenous background activity
CV(1)	Membrane capacitance

Supplementary Information: Demographic data

A wide selection of demographic data was collected from all participants:

- Age
- Sex
- Handedness
- Ethnicity
- MMSE score
- ACE score
- Years of education
- Highest level of education
- Previous occupation
- ‘Exercise’ score
- Alcohol intake
- Smoking habits
- ‘Social Network’ score
- ‘Travel’ score

The following information was collected from patients only:

- Days from diagnosis to scan
- Medical history
- Current and past medications
- Existing medical conditions

To calculate the ‘exercise’ score, participants were asked whether they would describe their current level of exercise as ‘sedentary’, ‘moderate’ or ‘vigorous’. Participants were given the score of one, two, or three, respectively. The ‘social network’ score was calculated as the sum of the number of close friends and close relatives the participant claimed to currently have. The ‘travel’ score was calculated as the sum of the number of times the participant claimed to travel out of the state per month, travel out of the country per year, whether they had ever lived out of the country (and how long for), and how frequently they currently travel per month.
